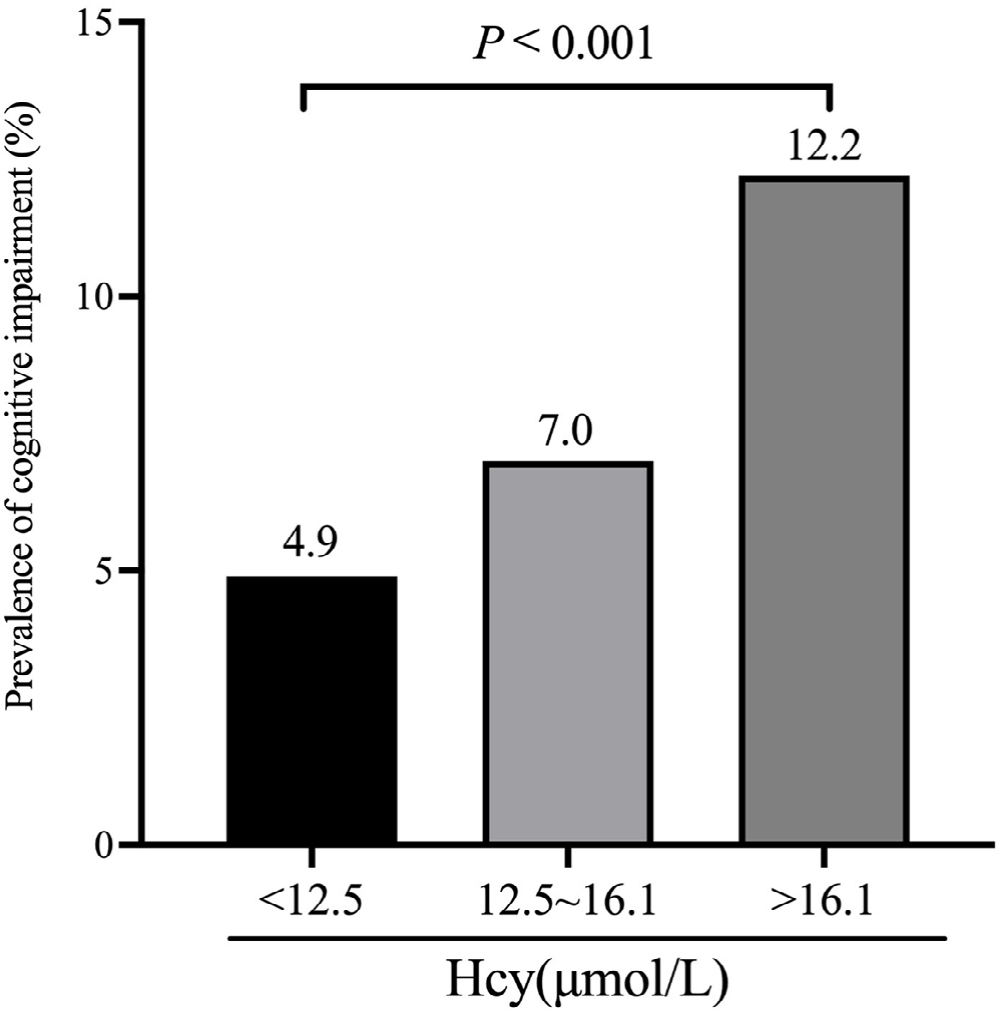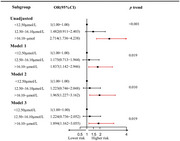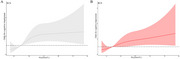# Plasma homocysteine levels are associated with cognitive impairment: a population‐based cross‐sectional study in a rural area of Xi’an, China

**DOI:** 10.1002/alz70860_101764

**Published:** 2025-12-23

**Authors:** Yi Zhao, Liangjun Dang, Suhang Shang, Shan Wei, Chen Chen, Jingyi Wang, Jin Wang, Qiumin Qu, Yan Qu

**Affiliations:** ^1^ The First Affiliated Hospital of Xi’an Jiaotong University, Xi’an, shaanxi, China; ^2^ The First Affiliated Hospital of Xi'an Jiaotong University, Xi'an, Shaanxi, China; ^3^ Huxian Hospital of Traditional Chinese Medicine, Xi'an, Shaanxi, China

## Abstract

**Background:**

High plasma homocysteine (Hcy) as a risk factor for cardiovascular disease and stroke has been known widely, however, the effects of plasma Hcy on cognitive impairment have not been determined fully. This study examined the relationship between plasma Hcy levels and cognitive impairment in a middle‐aged and older population in China.

**Method:**

This was a community population‐based cross‐sectional study using cluster‐sampling methodology. A total of 1,805 participants (≥ 40 years) were enrolled from a village in Xi’an, China. The global cognitive function was assessed by a Mini‐Mental State Examination (MMSE) and a battery of neuropsychological assessment scales, and cognitive impairment was diagnosed according to the criteria of mild cognitive impairment and dementia. Total plasma Hcy concentration was measured by the chemiluminometric assay. Plasma Hcy levels were categorized into three groups according to its tertiles:low‐level (Hcy<12.50μmol/L), moderate‐level (12.5≤Hcy ≤16.10μmol/L) and high‐level (Hcy>16.10μmol/L). Multivariable logistic regression analysis and subgroup analysis were conducted to investigate the relationship between Hcy and cognitive impairment. Non‐linear correlations were explored using restricted cubic splines.

**Result:**

Among 1,805 subjects, 145 (8.0%) met the cognitive impairment criteria. The prevalence of cognitive impairment was elevated across increasing tertiles of Hcy levels ( Tertile 1: 4.9%; Tertile 2: 7.0%; Tertile 3: 12.2%). Multivariable logistic regression showed that cognitive impairment incidence increased by 2.9% per unit increase in the Hcy level (OR=1.029, 95% CI: 1.011‐1.048, *p* = 0.001). Compared to the low‐level group, high‐level group had a significantly increased risk of cognitive impairment (*OR* =1.89, 95%CI: 1.16‐3.05, *p* = 0.010). Restricted cubic spline analysis did not find a non‐linear relationship (P_non‐linear_ =0.3167) between Hcy and cognitive impairment. Interaction analysis showed that sex, age, stroke history, diabetes, BMI, SBP did not affect the association between Hcy and cognitive impairment.

**Conclusion:**

Elevated plasma Hcy level is linked to an increased likelihood of cognitive impairment. However, the effects of Hcy on cognitive impairment need to be confirmed through reducing Hcy cohort intervention study.